# MaDREB1F confers cold and drought stress resistance through common regulation of hormone synthesis and protectant metabolite contents in banana

**DOI:** 10.1093/hr/uhac275

**Published:** 2022-12-07

**Authors:** Yi Xu, Wei Hu, Shun Song, Xiaoxue Ye, Zehong Ding, Juhua Liu, Zhuo Wang, Jingyang Li, Xiaowan Hou, Biyu Xu, Zhiqiang Jin

**Affiliations:** Haikou Experimental Station, Key Laboratory of Genetic Improvement of Bananas, Sanya Research Institute, State Key Laboratory of Biological Breeding for Tropical Crops, Institute of Tropical Bioscience and Biotechnology, Chinese Academy of Tropical Agricultural Sciences, Hainan, China; Hainan Key Laboratory for Protection and Utilization of Tropical Bioresources, Hainan Institute for Tropical Agricultural Resources, Chinese Academy of Tropical Agricultural Sciences, Hainan, China; Hainan Yazhou Bay Seed Laboratory, Hainan, China; Haikou Experimental Station, Key Laboratory of Genetic Improvement of Bananas, Sanya Research Institute, State Key Laboratory of Biological Breeding for Tropical Crops, Institute of Tropical Bioscience and Biotechnology, Chinese Academy of Tropical Agricultural Sciences, Hainan, China; Hainan Key Laboratory for Protection and Utilization of Tropical Bioresources, Hainan Institute for Tropical Agricultural Resources, Chinese Academy of Tropical Agricultural Sciences, Hainan, China; Hainan Yazhou Bay Seed Laboratory, Hainan, China; Hainan Key Laboratory for Biosafety Monitoring and Molecular Breeding in Off-Season Reproduction Regions, Key Laboratory of Biology and Genetic Resources of Tropical Crops, Institute of Tropical Bioscience and Biotechnology, Chinese Academy of Tropical Agricultural Sciences, Hainan, China; Haikou Experimental Station, Key Laboratory of Genetic Improvement of Bananas, Sanya Research Institute, State Key Laboratory of Biological Breeding for Tropical Crops, Institute of Tropical Bioscience and Biotechnology, Chinese Academy of Tropical Agricultural Sciences, Hainan, China; Hainan Yazhou Bay Seed Laboratory, Hainan, China; Haikou Experimental Station, Key Laboratory of Genetic Improvement of Bananas, Sanya Research Institute, State Key Laboratory of Biological Breeding for Tropical Crops, Institute of Tropical Bioscience and Biotechnology, Chinese Academy of Tropical Agricultural Sciences, Hainan, China; Hainan Key Laboratory for Protection and Utilization of Tropical Bioresources, Hainan Institute for Tropical Agricultural Resources, Chinese Academy of Tropical Agricultural Sciences, Hainan, China; Hainan Yazhou Bay Seed Laboratory, Hainan, China; Hainan Key Laboratory for Biosafety Monitoring and Molecular Breeding in Off-Season Reproduction Regions, Key Laboratory of Biology and Genetic Resources of Tropical Crops, Institute of Tropical Bioscience and Biotechnology, Chinese Academy of Tropical Agricultural Sciences, Hainan, China; Haikou Experimental Station, Key Laboratory of Genetic Improvement of Bananas, Sanya Research Institute, State Key Laboratory of Biological Breeding for Tropical Crops, Institute of Tropical Bioscience and Biotechnology, Chinese Academy of Tropical Agricultural Sciences, Hainan, China; Hainan Key Laboratory for Protection and Utilization of Tropical Bioresources, Hainan Institute for Tropical Agricultural Resources, Chinese Academy of Tropical Agricultural Sciences, Hainan, China; Hainan Yazhou Bay Seed Laboratory, Hainan, China; Hainan Key Laboratory for Biosafety Monitoring and Molecular Breeding in Off-Season Reproduction Regions, Key Laboratory of Biology and Genetic Resources of Tropical Crops, Institute of Tropical Bioscience and Biotechnology, Chinese Academy of Tropical Agricultural Sciences, Hainan, China; Haikou Experimental Station, Key Laboratory of Genetic Improvement of Bananas, Sanya Research Institute, State Key Laboratory of Biological Breeding for Tropical Crops, Institute of Tropical Bioscience and Biotechnology, Chinese Academy of Tropical Agricultural Sciences, Hainan, China; Hainan Key Laboratory for Protection and Utilization of Tropical Bioresources, Hainan Institute for Tropical Agricultural Resources, Chinese Academy of Tropical Agricultural Sciences, Hainan, China; Hainan Yazhou Bay Seed Laboratory, Hainan, China; Hainan Key Laboratory for Biosafety Monitoring and Molecular Breeding in Off-Season Reproduction Regions, Key Laboratory of Biology and Genetic Resources of Tropical Crops, Institute of Tropical Bioscience and Biotechnology, Chinese Academy of Tropical Agricultural Sciences, Hainan, China; Haikou Experimental Station, Key Laboratory of Genetic Improvement of Bananas, Sanya Research Institute, State Key Laboratory of Biological Breeding for Tropical Crops, Institute of Tropical Bioscience and Biotechnology, Chinese Academy of Tropical Agricultural Sciences, Hainan, China; Hainan Key Laboratory for Protection and Utilization of Tropical Bioresources, Hainan Institute for Tropical Agricultural Resources, Chinese Academy of Tropical Agricultural Sciences, Hainan, China; Hainan Yazhou Bay Seed Laboratory, Hainan, China; Hainan Key Laboratory for Biosafety Monitoring and Molecular Breeding in Off-Season Reproduction Regions, Key Laboratory of Biology and Genetic Resources of Tropical Crops, Institute of Tropical Bioscience and Biotechnology, Chinese Academy of Tropical Agricultural Sciences, Hainan, China; Haikou Experimental Station, Key Laboratory of Genetic Improvement of Bananas, Sanya Research Institute, State Key Laboratory of Biological Breeding for Tropical Crops, Institute of Tropical Bioscience and Biotechnology, Chinese Academy of Tropical Agricultural Sciences, Hainan, China; Key Laboratory of Hainan Province for Postharvest Physiology and Technology of Tropical Horticultural Products, South Subtropical Crops Research Institute, Chinese Academy of Tropical Agricultural Sciences, Guangdong, China; Haikou Experimental Station, Key Laboratory of Genetic Improvement of Bananas, Sanya Research Institute, State Key Laboratory of Biological Breeding for Tropical Crops, Institute of Tropical Bioscience and Biotechnology, Chinese Academy of Tropical Agricultural Sciences, Hainan, China; Hainan Key Laboratory for Protection and Utilization of Tropical Bioresources, Hainan Institute for Tropical Agricultural Resources, Chinese Academy of Tropical Agricultural Sciences, Hainan, China; Hainan Key Laboratory for Biosafety Monitoring and Molecular Breeding in Off-Season Reproduction Regions, Key Laboratory of Biology and Genetic Resources of Tropical Crops, Institute of Tropical Bioscience and Biotechnology, Chinese Academy of Tropical Agricultural Sciences, Hainan, China; Haikou Experimental Station, Key Laboratory of Genetic Improvement of Bananas, Sanya Research Institute, State Key Laboratory of Biological Breeding for Tropical Crops, Institute of Tropical Bioscience and Biotechnology, Chinese Academy of Tropical Agricultural Sciences, Hainan, China; Hainan Key Laboratory for Protection and Utilization of Tropical Bioresources, Hainan Institute for Tropical Agricultural Resources, Chinese Academy of Tropical Agricultural Sciences, Hainan, China; Hainan Yazhou Bay Seed Laboratory, Hainan, China; Hainan Key Laboratory for Biosafety Monitoring and Molecular Breeding in Off-Season Reproduction Regions, Key Laboratory of Biology and Genetic Resources of Tropical Crops, Institute of Tropical Bioscience and Biotechnology, Chinese Academy of Tropical Agricultural Sciences, Hainan, China

## Abstract

Adverse environmental factors severely affect crop productivity. Improving crop resistance to multiple stressors is an important breeding goal. Although *CBF*s*/DREB1*s extensively participate in plant resistance to abiotic stress, the common mechanism underlying *CBFs/DREB1*s that mediate resistance to multiple stressors remains unclear. Here, we show the common mechanism for *MaDREB1F* conferring cold and drought stress resistance in banana. *MaDREB1F* encodes a dehydration-responsive element binding protein (DREB) transcription factor with nuclear localization and transcriptional activity. *MaDREB1F* expression is significantly induced after cold, osmotic, and salt treatments. *MaDREB1F* overexpression increases banana resistance to cold and drought stress by common modulation of the protectant metabolite levels of soluble sugar and proline, activating the antioxidant system, and promoting jasmonate and ethylene syntheses. Transcriptomic analysis shows that MaDREB1F activates or alleviates the repression of jasmonate and ethylene biosynthetic genes under cold and drought conditions. Moreover, MaDREB1F directly activates the promoter activities of *MaAOC4* and *MaACO20* for jasmonate and ethylene syntheses, respectively, under cold and drought conditions. MaDREB1F also targets the *MaERF11* promoter to activate *MaACO20* expression for ethylene synthesis under drought stress. Together, our findings offer new insight into the common mechanism underlying *CBF/DREB1*-mediated cold and drought stress resistance, which has substantial implications for engineering cold- and drought-tolerant crops.

## Introduction

Adverse environmental factors, such as low temperature, drought, and salt, severely limit crop growth, development, and productivity. To combat these harsh stressors, plants have developed various complicated mechanisms for their protection, including the perception and transduction of stress signaling, regulation of gene expression, and physiological and metabolic changes [1, 2]. Of these, transcription factors (TFs) are crucial for regulating abiotic stress signaling and gene expression. The TFs of the APETALA2/ethylene-responsive factor (AP2/ERF) superfamily are subclassified into three families, namely ERF, AP2, and Related to ABI3/VP1 (RAV) [3]. The ERF family, which contains the ERF and DREB (dehydration-responsive element-binding protein) subfamilies, has a single AP2 domain and fewer introns and plays a key role in abiotic stress response [2]. Evidence has suggested the induction of *ERF*s by abiotic stress, such as drought, cold, heat, salt, and hormones, including abscisic acid (ABA), ethylene, gibberellin, salicylic acid, jasmonate (JA), and auxin, in various plant species [4]. Further genetic studies support the positive or negative function of ERFs in plant resistances to drought, cold, heat, salt, and heavy metals [4].

DREB TFs act on the activation of stress-responsive genes by directly binding to the C-repeat/dehydration responsive element (CRT/DRE) with a core sequence of G/ACCGAC, constituting the major component of the ABA-independent pathway during the stress response [2]. In *Arabidopsis*, DREBs are classified into six subclades, with subclade A1 mainly including C-repeat binding factors (CBF1–4) [5]. Extensive overexpression and silence studies have suggested that *CBF1* and *CBF3* play central roles in cold acclimation [6, 7]. In contrast, the function of *CBF2* is ambiguous; the T-DNA insertion mutation of *CBF2* increased freezing resistance [8], while the *cbf2* mutant was susceptible to freezing stress [9]. *CBF*s have been characterized in various plant species [10, 11] and overexpression of *CBF*s induces cold-responsive (*COR*) gene expression and increases cold resistance in some plant species, including rice, tomato, *Zea mays*, and *Brassica napus* [12–17]. Although this indicates the functional conservation of *CBF*s in cold resistance, other roles have been verified for diverse CBF members. *CBF4* plays a role in drought resistance and *CBF2* regulates dehydration and salt stress resistance in *Arabidopsis* [8, 18]. Overexpression of the orthologs (*OsDREB1A*, *OsDREB1B*, *OsDREB1E*, *OsDREB1F*, and *OsDREB1G*) of *Arabidopsis CBF*s increased rice resistance to drought and/or salt stress [19]. However, the common mechanism underlying *CBF/DREB1*-mediated multiple stress resistance remains unclear.

Accumulated evidence has shown that the crucial targets of CBFs/DREB1s are *COR* genes [6, 20, 21]. *COR* genes include *COR/LEA*, low-temperature induced (*LTI*), early dehydration-inducible (*ERD*), and responsive to desiccation (*RD*) genes [6, 20, 22–24]. The products of *COR* genes mainly include key enzymes involved in cell wall modifiers, osmolyte biosynthesis, lipid metabolism, carbohydrate metabolism, proteins for hormone responses, and molecular chaperones [6, 20, 21]. The promoters of *COR* genes harbor the CRT/DRE *cis*-element that is responsible for cold-, dehydration-, and salt-induced expression [20]. CBFs can regulate transcriptional changes in many genes associated with stress responses, TFs, kinases, hormonal signaling, carbohydrate metabolism, and cell wall modification [9, 25, 26]. However, in addition to *COR* genes, the target genes directly bound by CBF are still poorly understood.

Banana is one of the nutritional fruits and staple foods worldwide. It is widely distributed in tropical and subtropical developing countries, but these regions have high rates of abiotic stress and plant disease, severely decreasing the yield and quality of banana [27]. Moreover, banana plants are sensitive to cold, drought, and salt stress [28]. It is essential to study the mechanism underlying banana’s response to abiotic stress to enhance resistance to abiotic stress. Several genes, including *MusaPIP2;6*, *MusaPIP1;2*, *MusaNAC042*, *MusaWRKY71*, *MusaSAP1*, *MusaSNAC1*, *MaPIP2-7*, and *MaSIP2-1*, have been shown to have a positive effect on banana resistance to abiotic stress [28–35]. However, there is currently no evidence of the genetic function and molecular mechanism of the AP2/ERF subfamily in banana’s response to abiotic stress.

In this study, *MaDREB1F* overexpression increased banana resistance to cold and drought stress. Combining physiological and transcriptomic analyses, we discovered that *MaDREB1F* overexpression in common triggered the accumulation of soluble sugar and proline, activated antioxidant system, and promoted the synthesis of jasmonate and ethylene under cold and drought stress. The findings offer new insights into the common mechanism of *MaDREB1F* conferring cold and drought stress and will aid in breeding strategies to improve crop resistance to cold and drought stress.

## Results

### *MaDREB1F* encodes a DREB transcription factor

**Figure 1:**
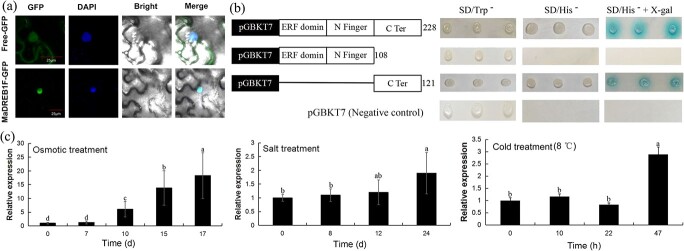
Subcellular localization, transactivation activity, and expression of *MaDREB1F* after drought, cold, and salt treatments. (a) Subcellular localization of *MaDREB1F*. 35S::*MaDREB1F*-GFP fusion and 35S::GFP were transiently transformed in tobacco leaves and visualized on the third day post-infiltration. (b) Transactivation activity of MaDREB1F in yeast. Fusion protein was transformed into the AH109 strain. Transactivation activity was examined on SD/Trp^−^, SD/His^−^, and SD/His^−^ with X-gal media. (c) Expression of *MaDREB1F* after osmotic, cold, and salt treatments. Duncan’s range test was used for significance examination (*n* = 3; *P* < .05).

Previously, our transcriptomic data indicated that *MaDREB1F* is induced after osmotic, cold, and salt treatments [36]. In this study, to investigate its role, we cloned the open reading frame (ORF) of *MaDREB1F*, which is 687 bp in length and encodes 229 amino acids. The BLASTX analysis showed that MaDREB1F has high homology with EgDREB1F (60%) from *Elaeis guineensis*, PdDREB1F (60%) from *Phoenix dactylifera*, and AcDREB1F (58%) from *Ananas comosus*. The putative MaDREB1F protein had typical sequence characteristics of a conserved AP2 domain, a nuclear localization signal (NLS), and an activation domain (Supplementary Data Fig. S1). Phylogenetic analysis showed that OsDREB1F belongs to the A-1 group of the DREB subfamily and is close to OsDREB1F (Supplementary Data Fig. S2). Subcellular localization analysis suggested that the fluorescence of 35S::GFP was distributed throughout the cell, whereas fluorescence of the 35S::*MaDREB1F*-GFP chimera was localized in the cellular nucleus (Fig. 1a). Transcriptional activity analysis showed that yeast cells containing pGBKT7-*MaDREB1F* and pGBKT7-*MaDREB1F-C* grew well in SD/His^−^ medium and turned blue in SD/His^−^ medium with X-gal, indicating transcriptional activity of MaDREB1F protein and its C-terminal domain (Fig. 1b). These results suggest that *MaDREB1F* encodes a DREB TF in banana.

### MaDREB1F expression is upregulated after osmotic, cold, and salt treatments

The expression of the *MaDREB1F* gene was induced by osmotic treatment and reached the highest level after 17 days of treatment. After salt treatment, the expression of *MaDREB1F* was slightly upregulated and reached the highest transcription level at 24 days. After cold treatment, *MaDREB1F* was induced at 47 hours of treatment (Fig. 1c). These results indicate the induction of *MaDREB1F* expression by osmotic, salt, and cold stress.

### Generation of transgenic banana plants overexpressing *MaDREB1F*

**Figure 2:**
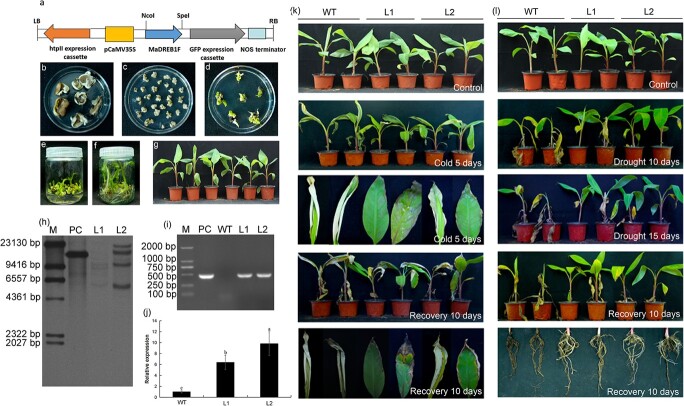
*MaDREB1F* overexpression increases banana resistance to cold and drought stress. (a) T-DNA region of pCAMBIA1302 for generating transgenic plants. (b) Floral apex slices on differentiation medium. (c) Callus regenerated from floral apex slices. (d) Induction of shoot growth on shooting medium. (e) Shoot growth to 3 cm in height. (f) Rooting of explants on rooting medium. (g) Hardening of rooted transgenic plants. (h) Integration of *MaDREB1F* transgene in lines L1 and L2 from Southern blot analysis. M, marker; PC, positive control. (i) Amplification of *hpt-II* gene by PCR in transgenic lines. (j) *MaDREB1F* expression in transgenic lines using qRT–PCR. Duncan’s range test was used for significance examination (*n* = 3; *P* < .05). (k) 100-day-old banana seedlings were exposed to cold conditions (8°C) for 5 days and recovery for 10 days, then photographs were taken. (i) 100-day-old banana seedlings were treated by withholding water for 10 and 15 days and recovery for 10 days, then photographs were taken.

To investigate the role of *MaDREB1F in planta*, *MaDREB1F* was introduced into the pCAMBIA1302 vector (Fig. 2a). The floral apices of the Gongjiao (*Musa corniculata* L. AAA group) were cut into slices that were subsequently placed on a differentiation medium to induce callus growth (Fig. 2b). Two-millimeter slices were cut from the callus and infected with *A. tumefaciens* EHA105 harboring the binary vector (Fig. 2c). The infected callus slices were transferred to a differentiation medium to induce shoot growth (Fig 2d). After the shoot had been cultured to 3 cm in height (Fig. 2e), the explants were placed in a rooting medium (Fig. 2f). When the roots reached 8 cm in length, the plantlets were moved onto coconut coir medium for 100 days (Fig. 2g). Finally, we acquired 27 hygromycin-resistant lines, of which six transgenic lines could be verified by PCR amplifying the *hpt-II* gene from the binary vector. Then, the *T*_2_ generation was produced from these transgenic lines. To provide molecular evidence for these transgenic lines, we performed a molecular characterization in detail. Southern blot analysis indicated the integration of multiple copies of the *MaDREB1F* transgene in lines L1 and L2 (Fig. 2h). In addition, PCR amplification indicated the existence of the *hpt-II* gene from the vector in L1 and L2 (Fig. 2i). The expression levels of *MaDREB1F* were 6–10 times higher in L1 and L2 than in the WT (Fig 2j). These results suggest that *MaDREB1F* was successfully overexpressed in banana plants.

### *MaDREB1F* overexpression enhances banana resistance to cold and drought stress

When banana seedlings were under cold and recovery conditions, WT plants displayed more severe leaf curling and chlorosis than transgenic lines (Fig. 2k). After 10 and 15 days of withholding water and 10 days of recovery, WT plants showed more severe growth inhibition, and leaf wilt and chlorosis compared with transgenic lines. Moreover, WT plants had shorter roots and more root damage than transgenic lines under drought and recovery conditions (Fig. 2l). *MaDREB1F* overexpression enhances banana’s resistance to cold and drought stress.

**Figure 3:**
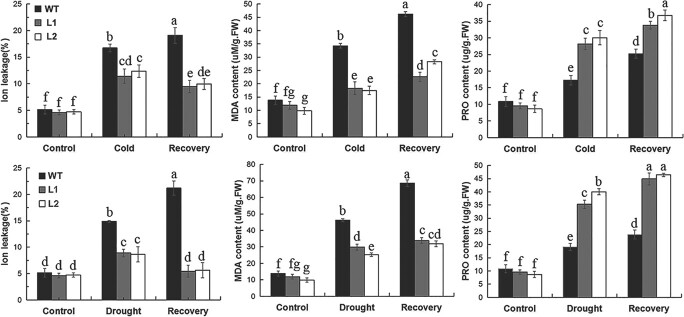
Physiological differences between transgenic lines and WT under normal, cold, drought, and recovery conditions. 100-day-old banana seedlings were exposed to low temperature at 8°C for 5 days and recovery for 10 days or treated by withholding water for 15 days and recovery for 10 days; leaf samples were then collected to examine physiological indices. Duncan’s range test was used for significance examination (*n* = 3; *P* < .05).

Under normal growth conditions, the content of MDA was lower in transgenic plants than in WT plants. Under drought, cold, and recovery conditions, transgenic plants exhibited lower levels of ion leakage and MDA, but higher proline content compared with WT plants (Fig. 3). Ion leakage and MDA are crucial indicators of reactive oxygen species (ROS)-mediated injury and membrane injury, respectively [37,38]. This further supports the positive role of *MaDREB1F* during drought and cold stress.

### Identification of cold- and drought-responsive genes affected by *MaDREB1F* overexpression

Comparative transcriptomic analysis was conducted between WT and *MaDREB1F-*overexpressing plants before and after drought and cold treatments. Because *MaDREB1F*-overexpressed plants were more tolerant than WT under drought and cold conditions, we focused on the differentially expressed genes (DEGs) that either specifically changed in *MaDREB1F*-overexpressing plants or changed ≥2-fold in *MaDREB1F*-overexpressing plants relative to the WT. Since these two types of DEGs are probably associated with stress resistance in banana conferred by *MaDREB1F*, we defined them as stress-responsive genes affected by *MaDREB1F* overexpression.

**Figure 4:**
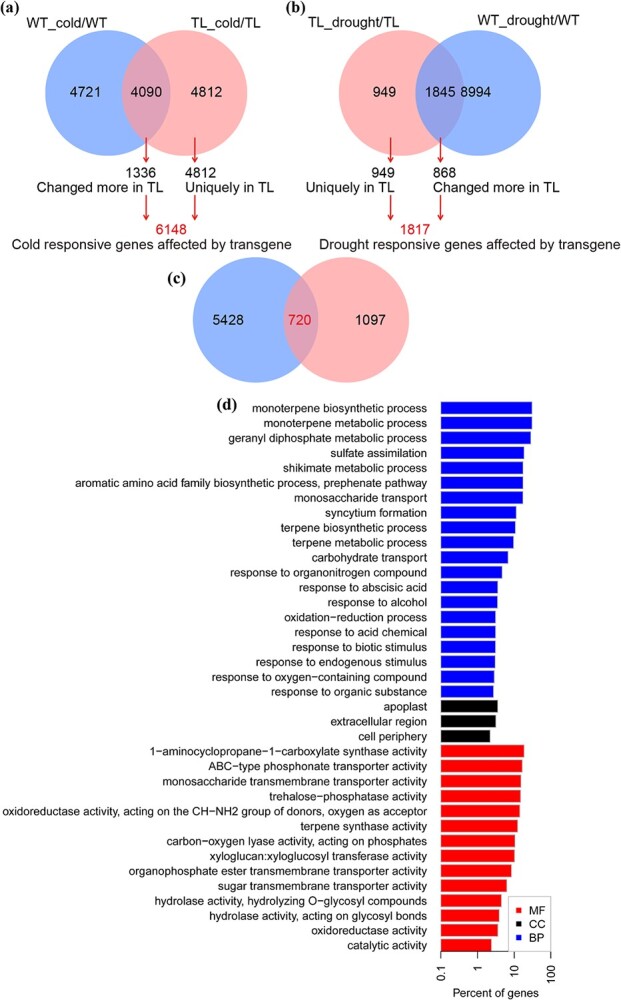
Identification of cold- and drought-responsive genes affected by *MaDREB1F* overexpression. (a) Venn diagram showing the cold-responsive genes in WT and the transgenic line. (b) Venn diagram showing the drought-responsive genes in WT and the transgenic line. (c) Venn diagram showing the genes regulated in common by *MaDREB1F* overexpression after cold and drought treatments. (d) GO enrichment of the genes regulated in common by *MaDREB1F* overexpression after cold and drought treatments.

After cold treatment, a total of 8902 and 8811 DEGs were identified from the transgenic line (TL)_cold/TL and WT_cold/WT, respectively (Supplementary Data Tables S2 and S3). Of these genes, 4812 were uniquely identified in TL_cold/TL, 4721 genes were exclusively found in WT_cold/WT, and 4090 genes were regulated in common in both WT_cold/WT and TL_cold/TL. Of the 4090 commonly regulated DEGs, 1336 genes changed more (fold change >2) in TL_cold/TL than in WT_cold/WT. In total, we identified 6148 genes as cold-responsive genes affected by *MaDREB1F* overexpression (Fig. 4a and Supplementary Data Table S4).

After drought treatment, a total of 10 839 and 2794 DEGs were identified from WT_drought/WT and TL_drought/TL, respectively (Supplementary Data Tables S5 and S6). Of these, 8994 genes were exclusively found in WT_drought/WT, 949 genes were uniquely identified in TL_drought/TL, and 1845 genes were commonly regulated in both WT_drought/WT and TL_drought/TL. Of these 1845 commonly regulated genes, 868 genes changed more (fold change >2) in TL_drought /TL than in WT_drought/WT. In total, 1817 genes were identified as drought-responsive genes affected by *MaDREB1F* overexpression (Fig. 4b and Supplementary Data Table S7).

We further overlapped the cold- and drought-responsive genes affected by *MaDREB1F* overexpression and found 720 genes that were commonly regulated by *MaDREB1F* overexpression after cold and drought treatment (Fig. 4c and Supplementary Data Tables S8 and S9). These genes belonged to 37 categories from the Gene Ontology (GO) enrichment analysis (Fig. 4d and Supplementary Data Table S10). Notably, these GO terms included carbohydrate transport (GO_0008643), sugar transmembrane transporter activity (GO_0051119), response to abscisic acid (GO_0009737), oxidoreductase activity (GO_0016491), oxidoreductase activity, acting on the CH-NH2 group of donors (GO_0016641), oxidation–reduction process (GO_0055114), and response to oxygen-containing compound (GO_1901700), suggesting that carbohydrate metabolism, ABA response, and oxidation–reduction-associated processes were commonly affected by *MaDREB1F* overexpression after cold and drought treatments (Supplementary Data Fig. S3 and Supplementary Data Tables S11 and S12). These seven GO terms harbor 146 genes and their expression patterns were classified into four groups. Of these, more genes (73 genes) in these biological processes showed common induction, but fewer genes (18 genes) showed common repression after cold and drought treatments in *MaDREB1F-*overexpressing plants than in the WT (Supplementary Data Fig. S4 and Supplementary Data Tables S13–S16).

**Figure 5:**
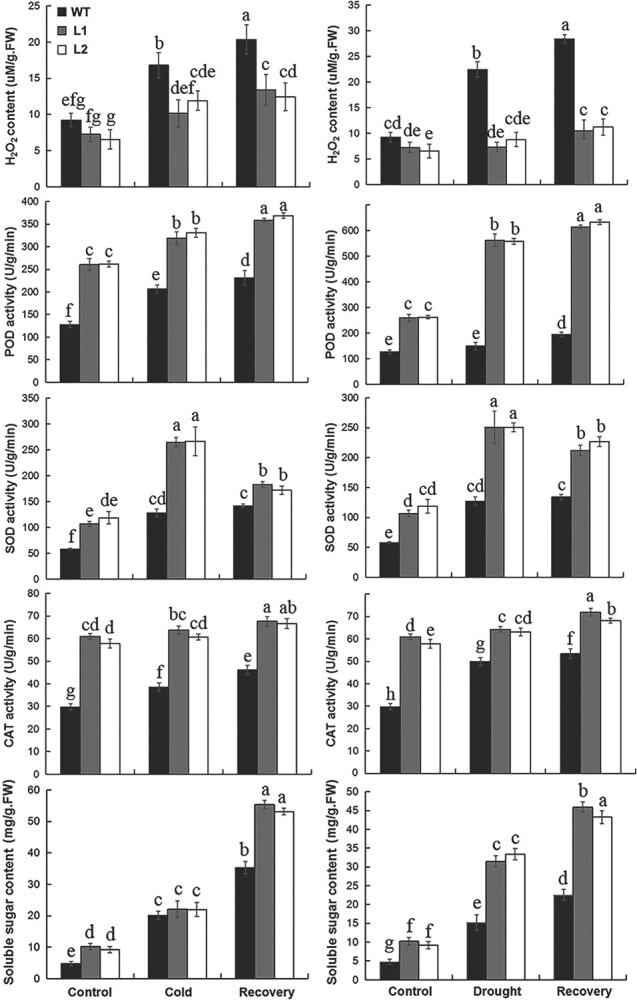
Measurement of H_2_O_2_ content, enzymatic activities of POD, SOD, and CAT, and soluble sugar content in WT and transgenic lines under normal, cold, drought, and recovery conditions. 100-day-old banana seedlings were exposed to low temperature at 8°C for 5 days and recovery for 10 days or treated by withholding water for 15 days and recovery for 10 days, then the leaf samples were collected to examine physiological indices. Duncan’s range test was used for significance examination (*n* = 3; *P* < .05).

Further physiological analyses showed that the H_2_O_2_ content was lower but activities of CAT, POD, and SOD and content of soluble sugar were higher in transgenic plants than in WT plants under normal, cold, drought, and recovery conditions. This indicates that *MaDREB1F* overexpression improves antioxidative enzyme activity and sugar metabolism, supporting the above transcriptomic results (Fig. 5).

### Common regulation of jasmonate and ethylene biosynthesis by *MaDREB1F* overexpression under cold and drought conditions

In the GO terms ‘response to oxygen-containing compound’ (GO_1901700) and ‘oxidation–reduction process’ (GO_0055114) from the commonly regulated gene set by *MaDREB1F* overexpression after cold and drought treatments, there are an *AOC* and two *ACOs* encoding key enzymes for biosynthesis of JA and ethylene, respectively (Supplementary Data Table S10). This guided us to investigate the effects of *MaDREB1F* on the JA and ethylene biosynthesis pathways under cold and drought conditions.

**Figure 6:**
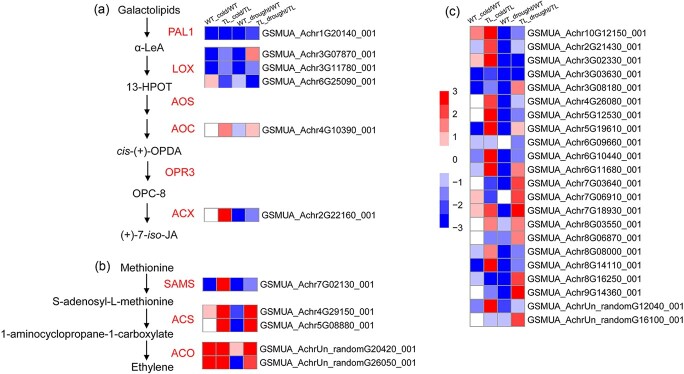
Common regulation of JA and ethylene biosynthesis by *MaDREB1F* overexpression under cold and drought conditions. (a) Expression profile of six genes commonly regulated by *MaDREB1F* overexpression after cold and drought treatments in the JA synthesis pathway. (b) Expression profile of five genes in the ethylene synthesis pathway commonly regulated by *MaDREB1F* overexpression after cold and drought treatments. (c) Expression profile of 22 ERF genes commonly regulated by *MaDREB1F* overexpression after cold and drought treatments.

We found that the expression of six genes encoding key enzymes in JA biosynthesis was significantly affected by *MaDREB1F* overexpression after cold and drought treatments (Fig. 6a and Supplementary Data Table S17). Under cold treatment, phospholipase A1 (*PAL1*) and two lipoxygenases (*LOX*s) (*GSMUA_Achr3G07870_001* and *GSMUA_Achr3G11780_001*) were repressed in WT and transgenic plants, but the degree of repression in transgenic plants was alleviated. Moreover, allene oxide cyclase (*AOC*) and acyl-CoA oxidase (*ACX*) did not show significant changes in the WT, but were significantly induced in transgenic plants under cold treatment. Under drought treatment, *PAL1*, *LOX* (*GSMUA_Achr3G11780_001*), and *ACX* were repressed in the WT, but the degree of repression was alleviated in *MaDREB1F-*overexpressing plants. In addition, another *LOX* (*GSMUA_Achr3G07870_001*) and *AOC* were repressed in WT plants, but were significantly induced in transgenic plants after drought treatment. These results indicated that *MaDREB1F* activated or alleviated the repression of genes in the JA biosynthesis pathway under cold and drought conditions.

The transcripts of five genes encoding ethylene biosynthesis-associated enzymes were commonly affected by *MaDREB1F* overexpression after cold and drought treatments (Fig. 6b and Supplementary Data Table S18). Ethylene biosynthesis includes three core enzymatic steps: *S*-adenosyl-l-methionine synthase (SAMS), ACS, and ACO. Under cold treatment, *SAMS* was downregulated and one *ACS* (*GSMUA_Achr5G08880_001*) did not show significant changes in WT, but they were significantly upregulated in *MaDREB1F-*overexpressing plants. One *ACS* (*GSMUA_Achr4G29150_001*) and two *ACOs* were upregulated in WT and transgenic plants, but the degree of induction was significantly improved in the transgenic plants. Under drought treatment, *SAMS* was repressed in WT and transgenic plants, but the degree of repression was alleviated in transgenic plants. Two *ACS* and one *ACO* (*GSMUA_AchrUn_randomG26050_001*) were repressed in WT plants, but were significantly induced in transgenic plants. The expression of one *ACO* (*GSMUA_AchrUn_randomG26050_001*) was upregulated in the WT and transgenic plants, but the degree of induction was significantly enhanced in the transgenic plants. These results indicate that *MaDREB1F* activated or alleviated the repression of genes in the ethylene biosynthesis pathway under cold and drought conditions.

Notably, we observed that expressions of 22 *ERFs* were commonly affected by *MaDREB1F* overexpression after cold and drought treatments. Under cold treatment, 15 *ERF*s showed more induction or less repression in transgenic plants than in WT. Similarly, 21 *ERF*s showed more induction or less repression in transgenic plants than in WT under drought treatment (Fig. 6c and Supplementary Data Table S19).

**Figure 7:**
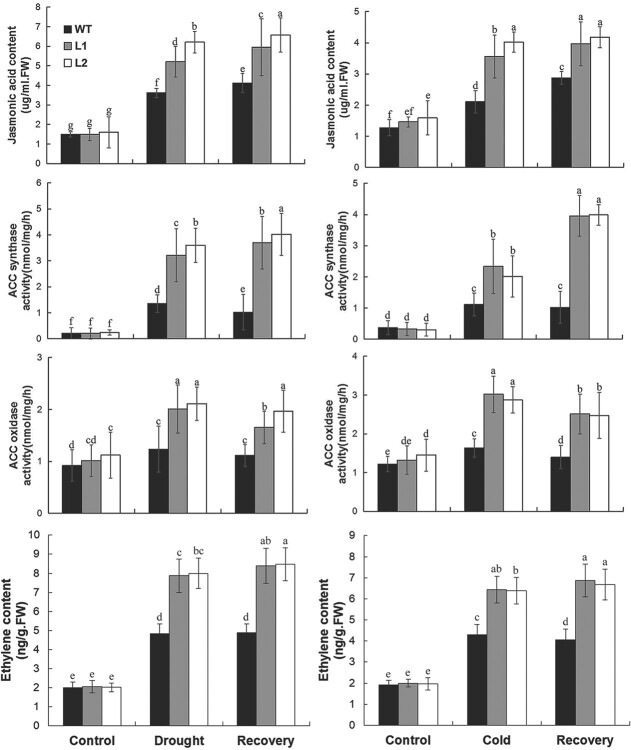
Measurement of JA content, ACS and ACO activities, and ethylene content in WT and transgenic lines cold, drought, and recovery conditions. 100-day-old banana seedlings were exposed to cold conditions (8°C) for 5 days and recovery for 10 days or treated by withholding water for 15 days and recovery for 10 days. The leaves were collected to examine JA content and activities of ACS and ACO. Whole seedlings were enclosed in an airtight container to collect and measure ethylene content. Duncan’s range test was used for significance examination (*n* = 3; *P* < .05).

Physiological analyses showed that JA content, ACS and ACO activities, and ethylene content were significantly higher in transgenic lines than in WT under cold, drought, and recovery conditions, further supporting the activation or alleviation of repression of JA and ethylene biosynthetic genes in transgenic plants under cold and drought conditions (Fig. 7).

### Transcriptional regulation of *MaAOC4*, *MaACO20*, and *MaERF11* by MaDREB1F

**Figure 8:**
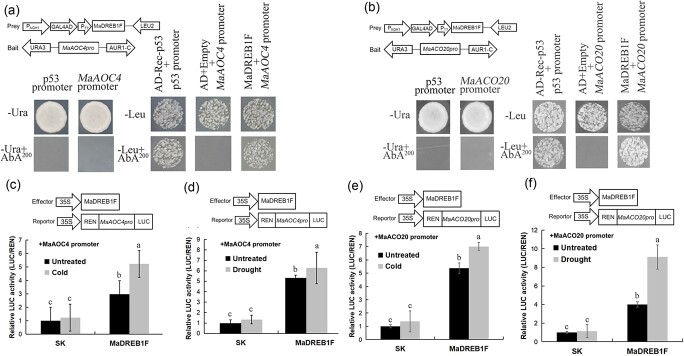
Direct activation of *MaAOC4* and *MaACO20* by MaDREB1F. (a, b) Physical interactions between MaDREB1F and *MaAOC4/MaACO20* promoters using a Y1H assay. Autoactivation of the promoters was examined on SD/−Ura + AbA medium, while the interactions between MaDREB1F and *MaAOC4/MaACO20* promoters were tested on SD/−Leu + AbA medium. (c–f) Analysis of relative LUC activity showed that MaDREB1F activates *MaAOC4/MaACO20* promoters under normal, cold, and drought conditions. Duncan’s range test was used for significance examination (*n* = 3; *P* < .05).

*MaAOC4* [*GSMUA_Achr4G10390_001*, belonging to response to oxygen-containing compound (GO_1901700)] and *MaACO20* [*GSMUA_AchrUn_randomG20420_001*, belonging to oxidation–reduction process (GO_0055114)] encode an allene oxide cyclase and a 1-aminocyclopropane-1-carboxylate oxidase, respectively, which are key enzymes for JA and ethylene biosynthesis. Because the promoter regions of these two genes contain the DRE/CRT *cis*-element (G/ACCGAC) (Supplementary Data Figs S5 and S6), the interactions of these promoters with MaDREB1F were detected using the Y1H assay. The results showed that yeast cells containing MaDREB1F-MaAOC4Pro and MaDREB1F-MaACO20Pro survived on the selective medium, suggesting interactions between MaDREB1F and the *MaAOC4* promoter and between MaDREB1F and the *MaACO20* promoter (Fig. 8a and b). To assess the effects of MaDREB1F on *MaAOC4/MaACO20* promoters, dual-luciferase assays were performed. Tobacco leaves that harbored MaDREB1F and luciferase (LUC) driven by *MaAOC4Pro* or *MaACO20Pro* showed significantly higher LUC activity than those that harbored SK vector and *LUC* driven by *MaAOC4Pro* or *MaACO20Pro* under normal, cold, or drought conditions (Fig. 8c–f). This indicates that MaDREB1F has an effect on activating *MaAOC4* and *MaACO20* promoters after cold and drought treatments.

**Figure 9:**
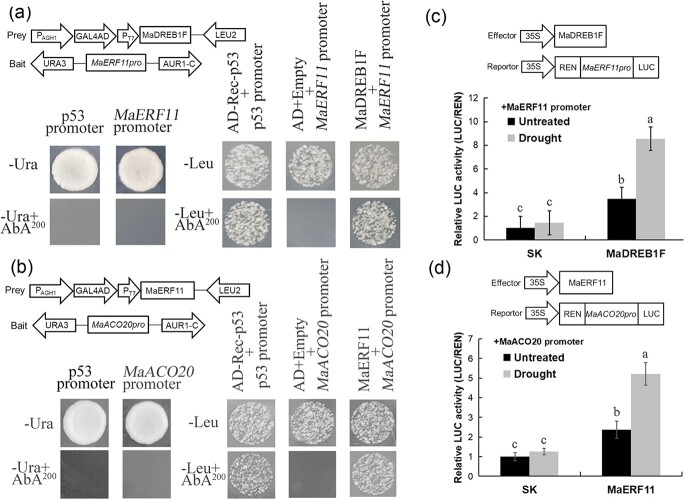
Direct activation of *MaERF11* by MaDREB1F and *MaACO20* by MaERF11. (a, b) Physical interactions between MaDREB1F and *MaERF11* promoter and between MaERF11 and *MaACO20* promoter using a Y1H assay. Autoactivation of the promoters was examined on SD/−Ura + AbA medium, while interactions between MaDREB1F and the *MaERF11* promoter and between MaERF11 and the *MaACO20* promoter were tested on SD/−Leu + AbA medium. (c, d) Relative LUC activity analysis showing that MaDREB1F activates the *MaERF11* promoter and MaERF11 activates the *MaACO20* promoter under normal and drought conditions. Duncan’s range test was used for significance examination (*n* = 3; *P* < .05).

Interestingly, an ethylene-responsive factor (*MaERF11*) exists in the gene set commonly regulated by *MaDREB1F* and the promoter region of *MaERF11* harbors the DRE/CRT *cis*-element. We thus investigated the interaction between *MaERF11* promoter and MaDREB1F using a Y1H assay and found that yeast cells containing MaDREB1F-MaERF11Pro survived on the selective medium, suggesting interaction between MaDREB1F and the *MaERF11* promoter (Fig. 9a). Moreover, the Y1H assay also indicated interaction between MaERF11 and the *MaACO20* promoter (Fig. 9b). Further dual-luciferase assays showed that MaDREB1F had an effect on activating the *MaERF11* promoter and that MaERF11 also activated the *MaACO20* promoter after drought treatment (Fig. 9c and d).

## Discussion

Improving crop resistance to multiple stresses is an important breeding goal. It is important to use the core functional genes for improving overall resistance of crops in molecular breeding [36]. Extensive studies have revealed that *DREB/CBF*s are critical for cold acclimation as well as resistance to drought and salt stress in higher plants [6, 19]. Currently, the common mechanism underlying *CBF/DREB1*-mediated multiple stress response remains unclear.

In this study we observed the transcriptional induction of *MaDREB1F* after cold, drought, and salt treatment, and *MaDREB1F* overexpression significantly enhanced banana resistance to cold and drought stress, in agreement with previous reports showing the positive effect of *CBF*s in the cold and drought responses of various plant species [12–19]. MaDREB1F has the typical characteristics of TFs supported by its nuclear localization and transcriptional activity. Due to the crucial function of TFs in regulating gene transcripts [1], further integrated transcriptomic analyses identified 720 genes that are commonly regulated by *MaDREB1F* overexpression after cold and drought treatments. Of these genes, those involved in carbohydrate metabolism, ABA response, and oxidation-reduction associated processes were affected in common by MaDREB1F overexpression after cold and drought treatments. Moreover, more genes (73 genes) in these biological processes showed common induction, but fewer genes (18 genes) showed common repression after cold and drought treatments in *MaDREB1F*-overexpressing plants than in WT, implying the possibly integrative activation of these biological processes by *MaDREB1F* overexpression after cold and drought treatments. Further physiological analyses support this hypothesis, by which H_2_O_2_ content decreased, but activities of CAT, POD, and SOD as well as the content of soluble sugar increased in transgenic plants under normal, cold, drought, and recovery conditions. This indicates that *MaDREB1F* overexpression improves antioxidant system and carbohydrate metabolism, and is thus beneficial for protecting transgenic plants against overproduced ROS and osmotic imbalance caused by cold and drought stress [39].

*MaDREB1F* has an effect on activating or alleviating repression of the genes in the JA and ethylene biosynthesis pathways under cold and drought conditions. This is further supported by physiological analyses showing the high JA contents and the activities of ACS and ACO in transgenic lines after cold and drought treatment. JA and ethylene are widely involved in stress responses in plants. There is evidence showing that exogenous JA and ethylene increased plant resistance to cold or drought stress [36,40–44]. Endogenous JA and ethylene levels increased under cold or drought stress [36,42,45,46]. Accordingly, JA and ethylene biosynthetic genes showed induction upon cold or drought stress [43,47–50]. Thus, coincident with the possible positive roles of JA and ethylene in cold and drought resistance, *MaDREB1F* confers cold and drought stress resistance through commonly activating or alleviating repression of JA and ethylene biosynthetic genes.

AOC, as a key enzyme in JA biosynthesis, participates extensively in plant resistance to various stress [51,52]. We found that MaDREB1F can directly bind to and activate the promoter of *MaAOC4* under cold and drought treatment, resulting in increased JA content in transgenic plants. The ICE-CBF transcriptional regulatory pathway plays a crucial role in plant response to cold stress. Upon cold stress, ICE1 and ICE2 are activated and directly bind to CANNTG *cis*-elements in the *CBF* promoters, leading to the induction of *COR* genes and increased cold resistance [36]. JA was reported to positively modulate the ICE-CBF pathway to improve cold resistance in *Arabidopsis* [53]. Moreover, the JA-induced ICE-CBF pathway also has a positive effect in resistance to cold stress of banana fruit [48]. Our results indicate that MaDREB1F can positively regulate the synthesis of JA through *MaAOC4* under cold stress. Thus, there may be a positive feedback loop, thereby improving plant resistance to cold stress.

ACO is a rate-limiting enzyme for ethylene biosynthesis [54]. Accompanied by an increase in ethylene production under cold and drought conditions, expression of *ACO*s significantly increased in various plant species [47,55–59]. We found that MaDREB1F can directly bind to and activate the promoter of *MaACO20* under cold and drought treatment. Additionally, MaDREB1F can also directly activate *MaERF11* expression and MaERF11 can directly activate *MaACO20* expression after drought treatment. This resulted in increased ACO activity and ethylene content in transgenic plants after cold and drought treatment. Although previous studies widely demonstrated the transcriptional response of the AP2/ERF superfamily (including CBFs) to ethylene upon abiotic stress [6,60], there is evidence showing that MaERF9 directly bound to and activated *MaACO1* promoter activity in banana fruit ripening [61]. Together, these findings reveal a multilayered regulatory cascade that promotes *MaACO20* transcription in the banana response to cold and drought stress.

**Figure 10:**
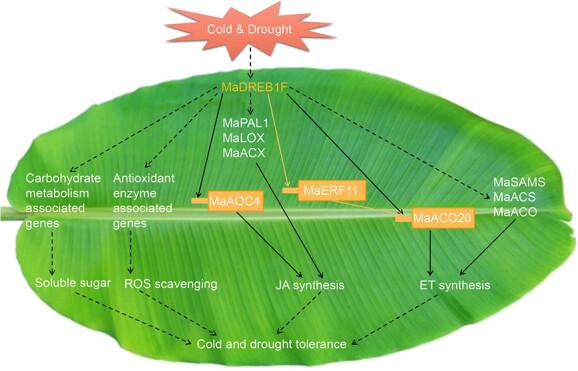
Proposed model for MaDREB1F conferring cold and drought stress resistance by commonly regulating hormone synthesis and protectant metabolite contents in banana. Solid black lines indicate a direct role, black dashed lines indicate an indirect role, and yellow lines indicate specific interactions for drought response.

Based on our present findings, we propose a possible working model for *MaDREB1F* conferring cold and drought stress resistance (Fig. 10). Upon cold and drought stress, MaDREB1F commonly affects carbohydrate metabolism- and antioxidant enzyme-associated genes, resulting in accumulation of soluble sugar and scavenging of ROS. Besides, MaDREB1F commonly activates or alleviates repression of *MaPAL1*, *MaLOX*s, *MaACX*, and *MaAOC4* for JA synthesis and of *MaERF11*, *MaSAMS*, *MaACO*s, and *MaACS*s for ethylene synthesis under cold and drought conditions. MaDREB1F directly activates promoter activities of *MaAOC4* and *MaACO20* for JA and ethylene synthesis, respectively, under cold and drought stress. Moreover, MaDREB1F can also target the *MaERF11* promoter to activate *MaACO20* expression for ethylene synthesis under drought stress. Our findings reveal a common mechanism underlying CBFs/DREB1-mediated resistance to cold and drought stress in plants and identify several new targets (*MaAOC4*, *MaACO20*, and *MaERF11*) of CBFs/DREB1s, beneficial for designing breeding strategies to improve crop resistance to cold and drought stress.

## Materials and methods

### Plant growth and stress treatment

Plants of the banana cultivar ‘Brazil’ (*Musa acuminata* L. AAA group) were used in this study. Seedlings were grown in a greenhouse (28°C, 16 hours light/8 hours dark cycle, 70% relative humidity). The stress treatments were applied on banana plants at the five-leaf stage. For cold treatment, the plants were placed in an incubator at 8°C for 47 hours with 70% relative humidity. For osmotic treatment, the plants were treated with 200 mM mannitol for 17 days at 28°C with 70% relative humidity. For NaCl treatment, plants were treated with 300 mM NaCl for 24 days at 28°C with 70% relative humidity.

### qRT–PCR

Banana leaves with or without cold, drought, and salt treatments were collected to examine *MaDREB1F* expression using qRT–PCR on a Stratagene Mx3000P instrument. The optimal primer and template concentrations were determined using a series of dilutions before the quantification experiments. Agarose gel electrophoresis and the melting curve were used to measure the specificity of the primers (Supplementary Data Table S1). Primer efficiency was examined in the range of 0.9–1.1. The expression of target genes was normalized using the comparative 2^–ΔΔCt^ method [62]. Expression of target genes was normalized with internal controls *MaUBQ2* (HQ853254) and *MaRPS2* (HQ853246).

### Cloning and subcellular localization of *MaDREB1F*

The coding sequence of *MaDREB1F* was amplified from the *M. acuminata* L. AAA group using RT–PCR (Supplementary Data TableS1). The PCR product was inserted into the pMD19-T vector and sequenced. The full-length cDNA sequence, which removed the stop codon, was inserted into the pCAMBIA1302 vector to produce an MaDREB1F:GFP fusion (Supplementary Data Table S1). *Agrobacterium tumefaciens* strain (GV3101, pSoup+p19) harboring the recombinant vector was independently infiltrated into the leaves of *Nicotiana benthamiana* by injection. After 3 days of culture, fluorescence was measured using a confocal laser-scanning microscope.

### Transcription activation activity of the MaDREB1F protein

The complete coding sequence and N- and C-terminals of *MaDREB1F* were cloned into the pGBKT7 vector (Supplementary Data Table S1). The constructs were transferred into yeast strain AH109, which was subsequently plated on plates with SD/Trp^−^ or SD/His^−^. Yeast cells were grown on medium containing X-gal to assess transcription activation activity.

### Generation of transgenic plants

Green fluorescent protein (GFP) as a gene expression reporter and vital marker has been widely used for studying plant development, gene expression regulation, and protein folding, and is not cytotoxic to plants [63–65]. The pCAMBIA1302-MaDREB1F-GFP construct was transferred into *Agrobacterium* strain GV3101. Overexpression of *MaDREB1F* in banana plants (*Musa corniculata* L. AAA group) was conducted using the *Agrobacterium-*mediated method [27]. Hygromycin-resistant transgenic plants were examined by amplifying the *hpt-II* gene from pCAMBIA1302. Then, the *T*_2_ generation was produced. Southern blot analysis was employed to further verify the integration of *MaDREB1F* in Line 1 (L1) and L2 genomes.

### Southern blotting assay

Total genomic DNA was extracted and digested with the EcoRI enzyme, followed by transfer to nylon membranes. The cDNA probe of *hpt-II* in pCAMBIA1302 was amplified with primer set 5′-GCTCCTACAAATGCCATCATTGC-3′ and 5′-GATAGTGGGATTGTGCGTCATCCC-3′ and labeled with a random primer labeling system. Hybridizations were conducted following the manufacturer’s instructions (Roche 11745832910, USA).

### Analyses of physiological indices

Ion leakage was tested following the method reported by Jiang and Zhang [66]. The content of malondialdehyde (MDA) was examined based on the thiobarbituric acid colorimetric method [67]. Proline content was examined following Bates *et al*. [68]. The content of H_2_O_2_ was measured with a detection kit (W-100, G-CLONE, Beijing, China). The activities of perox idase (POD), catalase (CAT) , and superoxide dismutase (SOD) were spectrophotometrically tested with detection kits (A007-1-1, A084-3-1, and A001-1-1; Jiancheng, Nanjing, China).. The soluble sugar content was examined using a detection kit (A145-1-1, Jiancheng, Nanjing, China). The content of jasmonate and the activities of 1-aminocyclopropane-1-carboxylic acid synthase (ACS) and 1-aminocyclopropane-1-carboxylic acid oxidase (ACO) were measured with detection kits (ml062281, ml062340-2, ml062341-2, Meilian, Shanghai, China). Banana seedlings were enclosed in an airtight container for 8 hours to collect ethylene. Ethylene production was examined by gas chromatography (GC-2010 Plus) following the manufacturer’s instructions.

### Transcriptomic analyses

Total RNA was extracted from the leaves of transgenic line L1 and wild type (WT) under normal, cold (5 days of cold treatment), or drought (10 days of drought treatment) conditions with an RNA extraction kit (EX1882, G-CLONE, Beijing, China). cDNA was converted from 3 μg RNA for each sample using the RevertAid First-Strand cDNA Synthesis Kit (Promega, Madison, WI, USA). Eighteen cDNA libraries were constructed and sequenced using Illumina GAII. After removing adapter sequences with the FASTX toolkit, FastQC was used to evaluate clean read quality. Assembly of the transcriptome was conducted using cufflinks with alignment files. Gene expression levels were represented as fragments per kilobase of transcript per million mapped reads (FPKM). DEGseq was employed to identify differentially expressed genes (DEGs) based on the threshold of 2-fold expression change and *P*-value ≤.05 of three replicate reads per gene.

### Yeast one-hybrid assay

In order to characterize the interaction between MaDREB1F and target promoters, each promoter sequence was constructed into pAbAi as a bait vector. The 2000-bp DNA sequences upstream of the transcription start site of the target genes were obtained from the banana A genome website (http://banana-genome.cirad.fr/). The 2000-bp fragment of the target promoter was used to construct the bait vector. The coding sequence of *MaDREB1F* was constructed into the pGADT7 as a prey vector (Supplementary Data Table S1). The suitable aureobasidin A (AbA) concentration for each bait vector is 200 mg/l. The prey and bait constructs were co-transformed into Y1H (yeast one-hybrid) yeast strains, followed by culture on SD/−Leu + AbA 200 selective medium at 30°C for 3 days.

### Dual-luciferase activity analysis

A dual-luciferase assay was conducted with the method by Niu *et al*. [69]. The promoter and coding sequence of the gene were cloned into pGreenII 0800-LUC and pGreenII 62-SK vectors, respectively, and were then transferred into *A. tumefaciens* GV3101 (Supplementary Data Table S1). Four-week-old tobacco leaves were infiltrated with the transformed *A. tumefaciens* using injection. Firefly luciferase (LUC) and *Renilla* luciferase (REN) activities were detected by a dual-luciferase reporter assay system. Three biological replicates were carried out for each assay.

## Acknowledgements

We thank the National Key Research and Development Program of China (2019YFD1000204), the 2020 Research Program of Sanya Yazhou Bay Science and Technology City (SKJC-2020-02-002), the National Natural Science Foundation of China (32260736), and the Central Public-interest Scientific Institution Basal Research Fund for Chinese Academy of Tropical Agricultural Sciences (1630052020006) for funding resources.

## Author contributions

W.H., B.X., and Z.J. conceived the study. Y.X. and W.H. wrote the manuscript. Other authors carried out experiments and analysis.

## Data availability

The data that support the findings are available in the paper and the supplementary materials published online.

## Conflict of interest

The authors declare no competing interests.

## Supplementary data

Supplementary data is available at *Horticulture Research* online.

## Supplementary Material

Web_Material_uhac275
