# Supplementary material for: MaDREB1F confers cold and drought stress resistance through common regulation of hormone synthesis and protectant metabolite contents in banana
Source: Hortic Res. 2022 Dec 7;10(2):uhac275. doi: 10.1093/hr/uhac275 (PMC9923210; doi:10.1093/hr/uhac275)
Supplement: Web_Material_uhac275 [file web_material_uhac275.zip › Supplementary Figures.docx]

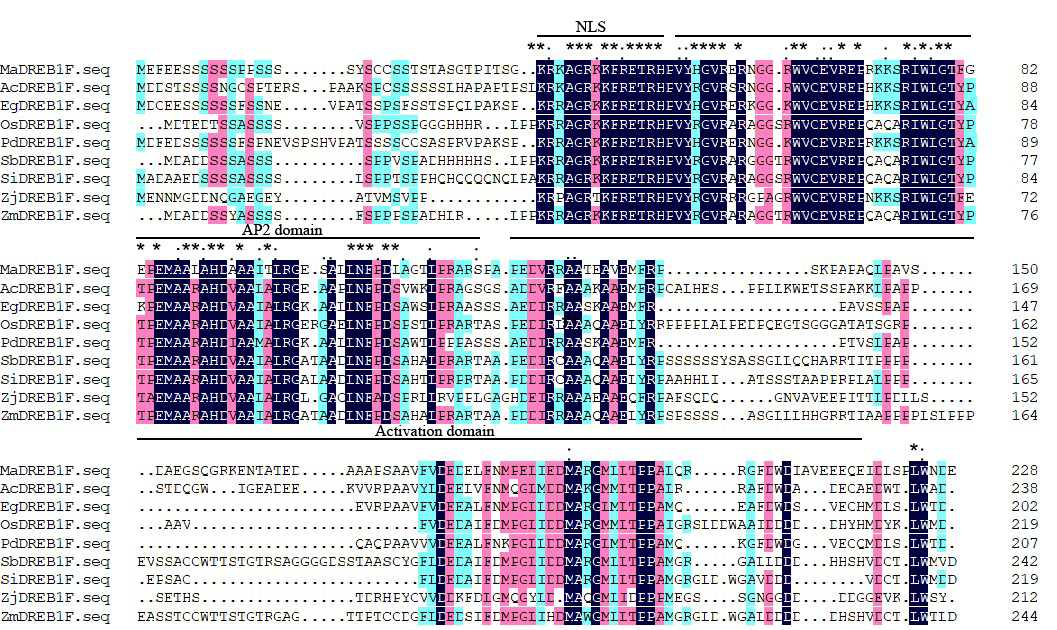


Figure S1. Sequence alignment of MaDREB1F with other known DREBF proteins including AcDREB1F (XM_020251774.1), EgDREB1F (XM_010942098.2), OsDREB1F (LK931476.1), PdDREB1F (XM_008809301), SbDREB1F (XM_002456971.2), SiDREB1F (XM_004971346.2), ZjDREB1F (AB627353.1) and ZmDREB1F (XM_008676258.2) using DNAMAN software. The lines marked show the nuclear localization signal (NLS), the conserved AP2 domain, and the activation domain. ‘‘*’’ indicates positions which have a single, fully conserved residue, ‘‘:’’ indicates highly conserved positions, and ‘‘.’’ indicates the weaker conserved positions.


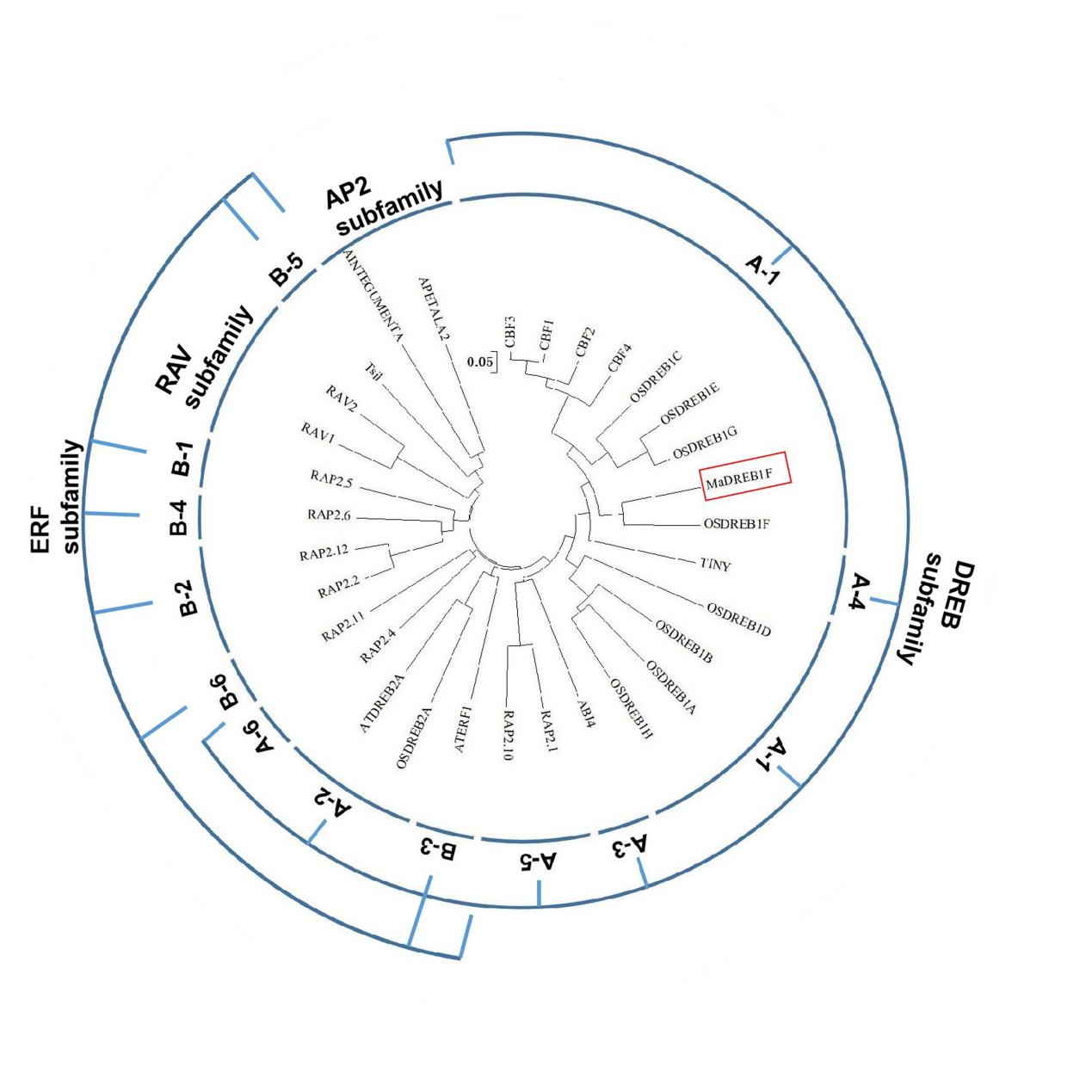


Figure S2. Phylogenetic analysis of MaDREB1F (red box) with other AP2/EREBPs from Arabidopsis and rice. The aligned members of the AP2 subfamily, RAV subfamily, DREB subfamily and ERF subfamily were the representative proteins of each subfamily. The phylogenetic tree was constructed with the full-length amino acid sequences by ClustalX 1.81 and MEGA 3.1 software.


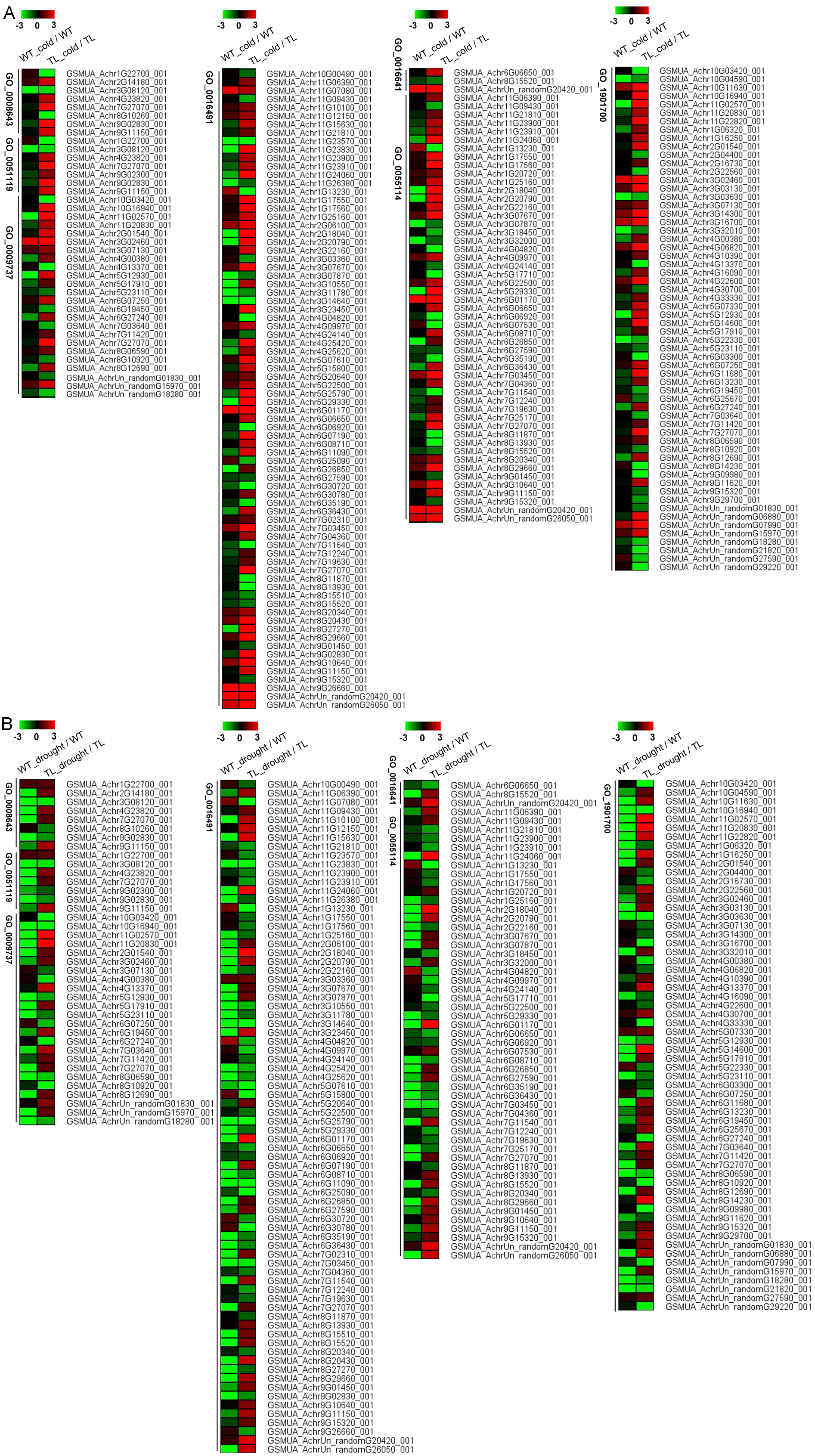


Figure S3. Expression profiles of the commonly regulated genes by *MaDREB1F* overexpression after cold and drought treatments in the 7 core GO terms. Log2 based fold changes was used to create the heat map. The scale represents the relative signal intensity of fold changes.


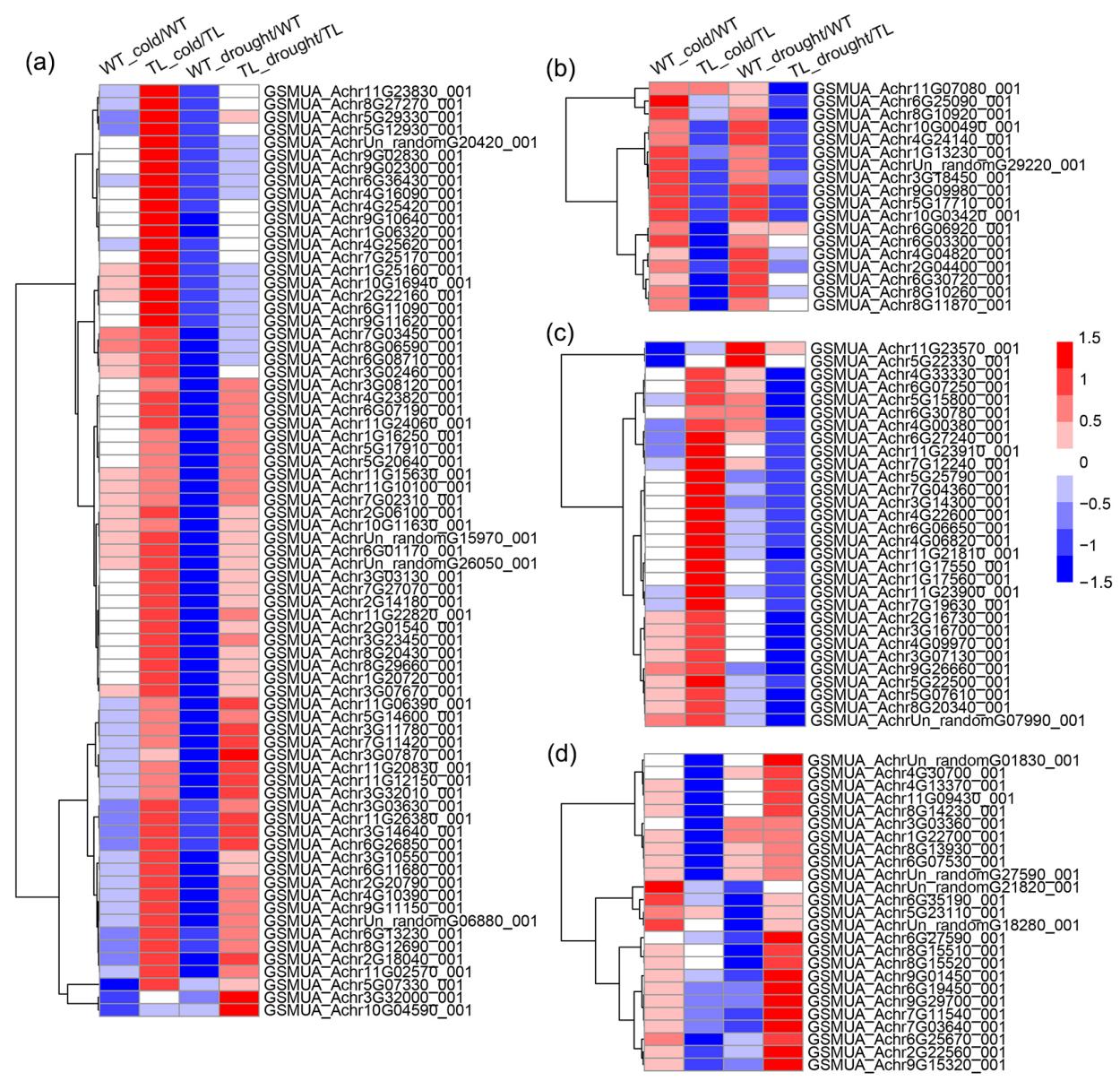


Figure S4. Expression of the 146 genes commonly regulated genes by *MaDREB1F* overexpression after cold and drought treatments in the 7 core GO terms. (a) (TL_cold / TL) > (WT_cold / WT) & (TL_drought / TL) > (WT_drought / WT) harboring 73 genes; (b) (TL_cold / TL) < (WT_cold / WT) & (TL_drought / TL) < (WT_drought / WT) harboring 18 genes; (c) (TL_cold / TL) > (WT_cold / WT) & (TL_drought / TL) < (WT_drought / WT) harboring 30 genes; and (d) (TL_cold / TL) < (WT_cold / WT) & (TL_drought / TL) > (WT_drought / WT) harboring 25 genes. Log2 based fold changes was used to draw the heat map. The scale represents the relative fold changes.

> GSMUA_Achr4G10390_001

TACCGCGGTGCGGCAAGATAAGCTCAGATTGCGGCGAGCAAAGGCGATGAAGCAAACGGTGGTTTTGTACGCAGTGCCGGCGATGGGCCACTTGGTGCCCATGGTGGAGCTGGCCAAGCTCTTCGTCCTCCACGACTTCTCCGTCGCTGTCGTCCTCATGCACACGCCCGTAAAACATCCCTCCGTGGACCCCTTCGTCGCCCGCGTCTCCTCCGCCTACCCCTCCATCTCCTTCCACCAGCTCCCGCCGGCCGCCTCACTCCCCGATACCCCGCTCCCCCGCTTCTTGGACCTCGTTCTCCCCAACAACCCCCAGCTCCTGTACTTCCTTGAAGCCCGTTCCCACACCTCCGACGTCCGCGCCGTCGTCCTCGACTTCTTCTGCACCGGTGCCCTGGCCGTCACCGCCAACCTCCGCCTCCCTTCCTACTTCTTCTTCGCCTCCTGCGCCGCCGTTCTCGCCGCCTTCCTCTATCTTCCGACCCTCTACGCCACCGCCGACATCGACTTAAAGGCCCTCGGGGACTCACCGCTCCACTTCCCAGGGCTGCCCCCCGTCCCCGCCTCCGACATGCCTCGCAACATGATCGACCGCGATGAAGACTACTTCAAGAGGATGATACGTGCCTTAGAGAGCCTGCCGAACGCCGACGGCATCCTGGTCAATTCGTTCGAGTCCCTGGAGGCGGAGGCCGTCCGGGTCCTTCGGGACGGGGCCTGCATTCCCGGTCGTCGGATGCCACCGGTTTACTGTATCGGGCCGTTGATCGCCGACGGGAGCAGGGACGTCGGAGGAGAAAAGATGGAGAAGGCTGAGTGCGCGTCGTGGTTGGACGAGCAACCGCGCGGGAGAGTAGTGTTCCTCTGCTTCGGTAGCATGGGGACGTTCTCCGCGGAGCAGCTCAAGGAGATCGCGGCTGGCCTGGAGAGGAGCGGACAGCGGTTCCTTTGGGTGGTGCGGGCTCCGCGGAGCGAGAGCCAGGGGCCGCAGGGATGGGGGCTGCAGTCAGAGCCGGACTTGGAGGCCCTGTTTCCGGAAGGCTTCTTGGAGCGGACAAAGCAGAGAGGATTCCTGGCGAAGTCGTGGGCACCGCAGGTGGAGGTGCTCAACCACGAGGCGGTGGGGGGGTTCGTAACGCACTGCGGGTGGAACTCGGTGCTGGAGGCGATCACGGCCGGGGTGCCGATGATCGGGTGGCCACTGTACGCGGAGCAGGGGATGAACAAGGTGCTCCTGGTGGAGCAGATGCGGGTGGCGGTGGCGATGGAGGGCTGCGCCAAGGAGCTGGTGGCGGCGGAGGAGGTTGAGGCCCGGATCAGGTGGCTGATGGAGTCGGAGGGGGGGCGGGACCTGAGGGCACGGGCGGTGGCGACGAAGCAGAGGGCGGCGGAGGCGATAAGGGAAGCCGGGTCGTCTCATCAGGCGTGGCTGGACGTAGTGAAGACCTTGAGGAATGGAAGCACGTCACCACTTCGAACCACAGGATTGACGAGTGAGGACCACCTCAAGGTGCCGTGCGATTGATGACATCCGCACCTGTCGGTTGAGGAGTAGTGGACTCCCCATGTGATGAAGCAATTCAATGGTTTGTGGACCTTTGTTGGAAAACCTAAAGATAAATATTAAAATATTTTGAATAAGTTAAAAATTTAGTCAAACAATTAAATTATTAATTAATTTATTTCTTAATAAATAATGTGATCCTTAAAAGTAAATAATTTAAATTTTATTTTTATATGTGAATAATAAAAAATATTATAATTTTACTTTACGTATAAAAGTAAAATAAAAATAAACTAAAAAATATATTATTGTTTACCTGAGCTAACTTTCAAAATTTTAAGAGCAGCTCATTTATTAGCTTATTAATTTTTATATGTATATTAATTGTTTTTTTCCTCGCTTCTTTTCCATATATTCAACCCACACCTTTCACCCCCCCGCCCACCCCGCCCCGCCCAAAAAAAAACACTAATAATAAAACGGAGTCTCCTCAAAC

Figure S5. The promoter sequence of *MaAOC4* (GSMUA_Achr4G10390_001). The

DRE/CRT *cis*-element (G/ACCGAC) are marked with yellow.

> GSMUA_AchrUn_randomG20420_001

GGTGGTGGAGAATCTCGCGGCGGTGGCGACCCCTGGAGTGGTGCCATCGATCATTAAGGTCTCTGCGTCTCTCTATCTCACTTCTCTTCTCTAAATGAACCAGCGAGGAATCTTGCAATCTCGATCCGGAAGAGATGCAGTTGATCGAAATGGGTTTTCTTTCGGTGCTTGGAGGTCTTCCTCTCCTTTCCCCTCCCTCCCTGCAGAAGGACGAGCGAGAAATGTTGTCGATTGACGCGGTGGTGACCCAACCCAATCCGAACCAACTCGAGTCTACTTGAACAAGCCCAACCCAGCCTCAATCATCTTAACTCGGATTGGGTTCGGCTCCAAATTGGCCCAACAATATTATAATGCCCACCAAATGATGTGTTGCAATTGCAATGCTGCGTGACTGACTTCAACAAACAAATGTTTGACCAGAGAGGCATGTGAAGTATTAGGAAAACCGCACATTAATTAAGTATACGAACGGCTCAGAAGAAAGCTACTCCTTCAGTCCCCCTACAATGTACTACTTGCCGTAGGAGTGACAACTACACTACAAAGCATTAATTGATGAACAACCAATCTGATCTCCAAATCCCACAGCAGATGATGTCCCATTAATATGATGTAGAATATCGCCATGATGTCGTAAAAAATAGATGCTCTGGTCTGTTCTTTCACGTATGATGGGAAAAATATCTATCTGATGGATTGAGTTCATTAAAGAATTCCTGTCCTCAATCTATTATGAGTTCTTGAAATGAGAGATTGGGCAATTTTTTAATCCATTCCTGCCCTTTTTTTTCATGACTGAATCATTACGGAAGACACTGAACCTAAACCATGGGGCAGTGAAGACATTGAACTCAGAGCAGCAACTGCACACTGCATCGGAGGAAGAGCTGCAGTGGACTAATGAATGATACACTCCAAGCCTAGAAGGTGTTTGCTTGTGGTTGGGTTTTTCTAGTTTACTGTCTCTTCAGCTGCTCTAGTTTCTTTTCGTGGCTTCTTGGATCCCAGTCTTCGATGAAGCACAAAGAATGAGTTCACGACGATGGAGACAGCAACCAAGGTGATGGGCACTTGGACCCCCCGGCAGAACTGGTGGCTGTATGCAGGATTAGGTAAGAGTTACTGTAGATGCATCCATTTGGCTTTCTTCTCTCCGTCAGATAATAAATGCAGTGAGGTAAGAGTAATGTATCTTTCTCTTCTCTGTTGGCTGCAGCTGTTCTTCAGAGGATGAGCTGTTTTGTGTGTGTATTCTGAAGACGTGTTACCAGTTTTGTGTGTCAGAAGCCTTATTTGACGTAGAAATTAATGATTTGTTCTTTAATCCACCGAAGTCATCTCTGCAGAAGATGAAGGGATATCATTGTTTGGAAGCGGAACAAGTCAAGAGGGCCGGGGGGGTGGGGGGGGGGGGATGCGGTTTGGGACACATGAGAAGTCGGCAGCGGGATGCTCGGACAACTGCCAACATGAACACCGAATGCAGCAACCTCAAGATGCTGTGAATGATCAAATGTTACGTTTCGATGAGGGCCTCAGAAGCACCAATAATTAATGGTCTATCTCATGTGACCACTGCAGATGTGCAGCTACCTTTCAGATGGCTACAACAAGCAACAACACTTGTTCCCATGCAGGGTGGAGAAGAGACGTGAGGGGCTGTCAGCGGTGTCACTGATGCGTAGGACAGAGCGGCATGGATGTGCATGACAAAGGGAATGGTCCAGCTCTGTTAGTGTTCGTGAGTTATGGGGGTGATGATGATGGAGTCTGTGACATGATCAGACGACCAGTGCACGTTCAATGCATTCATTCACTTGCCGACTCATGGAGCAGATTAAGAACAGCGGCCGAAATGATGATCGGGTTTCCTTTAGATATAAACTAGTAGTTGACATCCACGATGTTTGCACTATAAAAACCCCCCCAGAGGCCTTCCATTCCTTCACATCCATTGCAGAAGGGTGTTTCCCAGTCTTCTGTAGCTTTGAGGCATC

Figure S6. The promoter sequence of *MaACO20* (GSMUA_AchrUn_randomG20420_001). The DRE/CRT *cis*-element (G/ACCGAC) are marked with yellow.
